# Santal monohydrate, an isoflavone isolated from *Wye­thia mollis*

**DOI:** 10.1107/S1600536814002670

**Published:** 2014-02-08

**Authors:** Kyle S. Knight, Cole T. Smith, Thomas G. Waddell, Bruce Noll

**Affiliations:** a Department of Chemistry, The University of Tennessee at Chattanooga, Chattanooga, TN 37403, USA; b Crystallographic Systems, Bruker AXS Inc., 4565 East Cheryl Parkway, Madison, WI 53711, USA

## Abstract

The title compound [systematic name: 3-(3,4-di­hydroxy­phen­yl)-5-hy­droxy-7-meth­oxy-4*H*-chromen-4-one monohydrate], C_16_H_12_O_6_·H_2_O, is a monohydrate of a natural product santal isolated from *Wye­thia mollis*. In the santal mol­ecule, the dihedral angle between the benzo­quinone and di­hydroxy­phenyl fragments is 53.9 (1)° and an intra­molecular O—H⋯O hydrogen bond occurs. In the crystal, O—H⋯O hydrogen bonds link the components into corrugated layers parallel to the *ac* plane. The short distance of 3.474 (5) Å between the centroids of the benzene rings in neighbouring santal mol­ecules reveals then existence of π–π inter­actions within the layers.

## Related literature   

For the discovery and structural identification of isoflavones, see: Raudnitz & Perlmann (1935 ▶); Robertson *et al.* (1949 ▶). Santal was isolated following the method of Waddell *et al.* (1982 ▶). For the structure of the triterpene component of *Wye­thia mollis*, see: Smith *et al.* (2013 ▶).
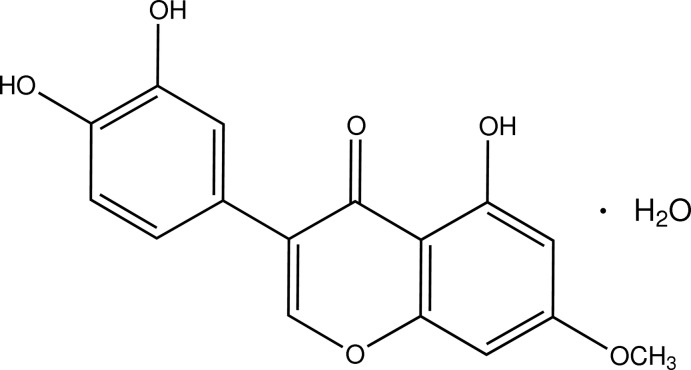


## Experimental   

### 

#### Crystal data  

C_16_H_12_O_6_·H_2_O
*M* *_r_* = 318.28
Orthorhombic, 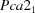

*a* = 16.494 (3) Å
*b* = 13.082 (3) Å
*c* = 6.6008 (12) Å
*V* = 1424.3 (5) Å^3^
*Z* = 4
Mo *K*α radiation
μ = 0.12 mm^−1^
*T* = 200 K
0.46 × 0.41 × 0.4 mm

#### Data collection  

Bruker APEXII CCD diffractometer
Absorption correction: multi-scan (*SADABS*; Sheldrick, 2004 ▶) *T*_min_ = 0.518, *T*_max_ = 0.958
8545 measured reflections
2478 independent reflections
2002 reflections with *I* > 2σ(*I*)
*R*_int_ = 0.060

#### Refinement  

*R*[*F* ^2^ > 2σ(*F* ^2^)] = 0.039
*wR*(*F* ^2^) = 0.110
*S* = 0.85
2478 reflections
221 parameters
3 restraints
H atoms treated by a mixture of independent and constrained refinement
Δρ_max_ = 0.14 e Å^−3^
Δρ_min_ = −0.18 e Å^−3^

### 

Data collection: *APEX2* (Bruker, 2009 ▶); cell refinement: *SAINT* (Bruker, 2009 ▶); data reduction: *SAINT* (Bruker, 2009 ▶); program(s) used to solve structure: *SHELXS97* (Sheldrick, 2008 ▶); program(s) used to refine structure: *SHELXS97* (Sheldrick, 2008 ▶); molecular graphics: *OLEX2* (Dolomanov *et al.*, 2009 ▶); software used to prepare material for publication: *OLEX2*.

## Supplementary Material

Crystal structure: contains datablock(s) I. DOI: 10.1107/S1600536814002670/cv5443sup1.cif

Structure factors: contains datablock(s) I. DOI: 10.1107/S1600536814002670/cv5443Isup2.hkl

Supporting information file. DOI: 10.1107/S1600536814002670/cv5443Isup3.cml

CCDC reference:

Additional supporting information:  crystallographic information; 3D view; checkCIF report

## Figures and Tables

**Table 1:** Hydrogen-bond geometry (Å, °)

*D*—H⋯*A*	*D*—H	H⋯*A*	*D*⋯*A*	*D*—H⋯*A*
O1—H1⋯O4^i^	0.84	1.98	2.777 (4)	157
O4—H4⋯O1*S*	0.84	1.88	2.708 (4)	169
O1*S*—H1*SA*⋯O5^ii^	0.91 (3)	1.97 (3)	2.855 (4)	164 (5)
O6—H6⋯O5	0.87 (4)	1.76 (4)	2.577 (4)	155 (4)

Symmetry codes: (i) 
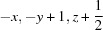
; (ii) 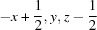.

**Fig. 1.**
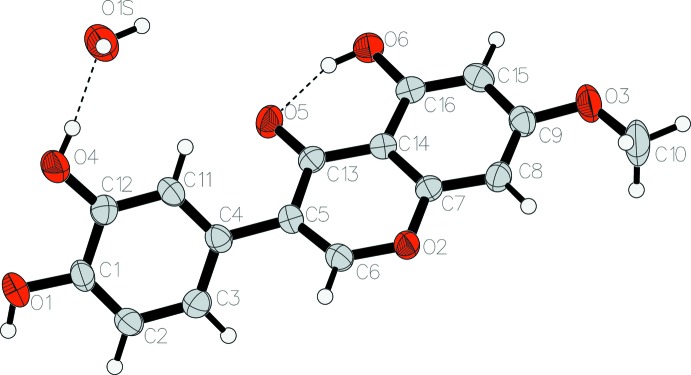
View of the title compound showing the atomic numbering and 50% probability displacement ellipsoids. Dashed lines denote hydrogen bonds.

## supplementary crystallographic information

### 1. Comment

Santal, C_16_H_12_O_6_, is an isoflavone isolated from *Wyethia mollis*, a species once used in folk medicine to treat contusions, pain, fevers, and colds. Santal (Figure 1), has a benzoquinone core with an appended dihydroxyphenyl group. The benzoquinone core is substituted with hydroxyl and methoxy substituents. In the santal molecule of the title compound, which is a monohydrate, the flat planes created by the benzoquinone core and the dihydroxyphenyl group are twisted dramatically relative to each other with a dihedral angle of 53.9 (1)°. The torsion angle C11—C4—C5—C13 is 54.1 (5)°. This twisting breaks conjugation between the rings, but is likely necessitated by steric interactions between O5 and H11.

The molecule stacks together with the benzoquinone rings parallel to each other and with the dihydroxyphenyl rings pointing in toward the center of the unit cell. The crystal structure shows the presence of linking external water molecules. The water interacts uniquely with three separate santal molecules. It acts as a hydrogen bond donor (H1SA) with O5 and as a hydrogen bond acceptor with O4H of a second santal molecule (Table 1). The second hydrogen on the water (H1SB) is stabilized by interaction with the electron rich π system of the dihydroxyphenyl ring of a third santal molecule. Additionally, O4 acts as a hydrogen bond acceptor to O1H in another santal unit. There is an intramolecular hydrogen bond in which the hydroxyl group at O6 acts as the donor and O5 as the acceptor (Table 1).

In the crystal, intermolecular O—H···O hydrogen bonds link all moieties into corrugated layers parallel to *ac* plane. The short distances of 3.474 (5) Å between the centroids of benzene rings from the neighbouring santal molecules reveal an existence of π–π interactions inside the layers.

### 2. Experimental

Santal was isolated as described previously (Waddell *et al.*, 1982). Suitable crystals of the title compound were obtained by slow evaporation of a water solution of the santal.

### 3. Refinement

H6 was located in a difference Fourier map and refined freely. H1SA and H1SB (H_2_O) were located in a difference Fourier map and refined with O—H distance restrained to 0.91 (3) Å, with *U*_iso_(H)= 1.5U_eq_ (O). All other H atoms were positioned geometrically, with bond distances of 0.85 Å for hydroxyl, 0.98 Å for methyl and 0.95 Å for those bound to aromatic rings and were refined as riding, with *U*_iso_(H)= 1.2–1.5U_eq_ of the parent atom.

### Crystal data

**Table:**

C_16_H_12_O_6_·H_2_O	*D*_x_ = 1.484 Mg m^−^^3^
*M**_r_* = 318.28	Mo *K*α radiation, λ = 0.71073 Å
Orthorhombic, *P**c**a*2_1_	Cell parameters from 2745 reflections
*a* = 16.494 (3) Å	θ = 2.5–24.8°
*b* = 13.082 (3) Å	µ = 0.12 mm^−^^1^
*c* = 6.6008 (12) Å	*T* = 200 K
*V* = 1424.3 (5) Å^3^	Prism, yellow
*Z* = 4	0.46 × 0.41 × 0.4 mm
*F*(000) = 664	

### Data collection

**Table:**

Bruker APEXII CCD diffractometer	2002 reflections with *I* > 2σ(*I*)
Graphite monochromator	*R*_int_ = 0.060
φ and ω scans	θ_max_ = 25.0°, θ_min_ = 2.5°
Absorption correction: multi-scan (*SADABS*; Sheldrick, 2004)	*h* = −18→19
*T*_min_ = 0.518, *T*_max_ = 0.958	*k* = −14→15
8545 measured reflections	*l* = −7→7
2478 independent reflections	

### Refinement

**Table:**

Refinement on *F*^2^	3 restraints
Least-squares matrix: full	H atoms treated by a mixture of independent and constrained refinement
*R*[*F*^2^ > 2σ(*F*^2^)] = 0.039	*w* = 1/[σ^2^(*F*_o_^2^) + (0.0829*P*)^2^] where *P* = (*F*_o_^2^ + 2*F*_c_^2^)/3
*wR*(*F*^2^) = 0.110	(Δ/σ)_max_ < 0.001
*S* = 0.85	Δρ_max_ = 0.14 e Å^−^^3^
2478 reflections	Δρ_min_ = −0.18 e Å^−^^3^
221 parameters	

### Special details

**Table:**

Geometry. All e.s.d.'s (except the e.s.d. in the dihedral angle between two l.s. planes) are estimated using the full covariance matrix. The cell e.s.d.'s are taken into account individually in the estimation of e.s.d.'s in distances, angles and torsion angles; correlations between e.s.d.'s in cell parameters are only used when they are defined by crystal symmetry. An approximate (isotropic) treatment of cell e.s.d.'s is used for estimating e.s.d.'s involving l.s. planes.

### Fractional atomic coordinates and isotropic or equivalent isotropic displacement parameters (Å^2^)

**Table:**

	*x*	*y*	*z*	*U*_iso_*/*U*_eq_	
O1	0.00097 (18)	0.5170 (2)	0.8482 (5)	0.0495 (7)	
H1	−0.0315	0.4972	0.9380	0.074*	
O2	0.05990 (13)	1.09967 (17)	1.0156 (4)	0.0349 (6)	
O3	0.25286 (16)	1.36978 (18)	1.0531 (5)	0.0496 (7)	
O4	0.10095 (17)	0.6010 (2)	0.5878 (4)	0.0519 (8)	
H4	0.1343	0.6336	0.5166	0.078*	
O5	0.24167 (15)	0.88921 (18)	0.9502 (4)	0.0410 (6)	
O6	0.34600 (14)	1.0348 (2)	0.9842 (4)	0.0387 (6)	
C1	0.0235 (2)	0.6152 (3)	0.8869 (6)	0.0359 (8)	
C2	−0.0036 (2)	0.6707 (3)	1.0506 (6)	0.0391 (8)	
H2	−0.0399	0.6403	1.1448	0.047*	
C3	0.0222 (2)	0.7715 (3)	1.0786 (5)	0.0380 (8)	
H3	0.0036	0.8090	1.1928	0.046*	
C4	0.0746 (2)	0.8173 (2)	0.9417 (5)	0.0316 (7)	
C5	0.1003 (2)	0.9250 (2)	0.9674 (5)	0.0306 (7)	
C6	0.0442 (2)	0.9991 (3)	0.9915 (5)	0.0335 (7)	
H6A	−0.0111	0.9788	0.9914	0.040*	
C7	0.13907 (18)	1.1317 (2)	1.0132 (4)	0.0282 (7)	
C8	0.1518 (2)	1.2363 (3)	1.0322 (5)	0.0339 (7)	
H8	0.1077	1.2826	1.0432	0.041*	
C9	0.2307 (2)	1.2700 (3)	1.0344 (5)	0.0343 (8)	
C10	0.1904 (3)	1.4455 (3)	1.0619 (9)	0.0680 (14)	
H10A	0.1572	1.4416	0.9390	0.102*	
H10B	0.2150	1.5135	1.0718	0.102*	
H10C	0.1562	1.4332	1.1808	0.102*	
C11	0.1018 (2)	0.7603 (3)	0.7752 (5)	0.0342 (8)	
H11	0.1380	0.7905	0.6808	0.041*	
C12	0.0764 (2)	0.6607 (3)	0.7470 (5)	0.0356 (8)	
C13	0.18522 (19)	0.9544 (2)	0.9687 (4)	0.0284 (7)	
C14	0.20191 (18)	1.0609 (2)	0.9938 (4)	0.0271 (7)	
C15	0.29629 (19)	1.2025 (3)	1.0181 (5)	0.0350 (8)	
H15	0.3503	1.2278	1.0207	0.042*	
C16	0.28220 (19)	1.1001 (2)	0.9983 (5)	0.0297 (7)	
O1S	0.1966 (2)	0.6972 (2)	0.3142 (5)	0.0571 (8)	
H1SA	0.225 (3)	0.755 (3)	0.345 (9)	0.086*	
H1SB	0.162 (3)	0.711 (4)	0.214 (7)	0.086*	
H6	0.323 (2)	0.975 (3)	0.975 (6)	0.044 (12)*	

### Atomic displacement parameters (Å^2^)

**Table:**

	*U*^11^	*U*^22^	*U*^33^	*U*^12^	*U*^13^	*U*^23^
O1	0.0547 (17)	0.0299 (15)	0.0639 (18)	−0.0135 (12)	0.0080 (13)	−0.0013 (13)
O2	0.0262 (12)	0.0265 (12)	0.0520 (13)	0.0019 (10)	0.0011 (11)	−0.0010 (11)
O3	0.0511 (16)	0.0283 (14)	0.0694 (18)	−0.0072 (13)	−0.0025 (14)	−0.0057 (13)
O4	0.0612 (19)	0.0411 (17)	0.0534 (16)	−0.0198 (13)	0.0149 (13)	−0.0138 (12)
O5	0.0322 (13)	0.0305 (14)	0.0602 (16)	0.0054 (11)	−0.0029 (10)	−0.0060 (12)
O6	0.0266 (12)	0.0382 (15)	0.0513 (15)	0.0031 (11)	−0.0018 (11)	−0.0011 (11)
C1	0.0334 (19)	0.0228 (19)	0.051 (2)	−0.0029 (14)	−0.0024 (15)	0.0019 (15)
C2	0.0356 (19)	0.031 (2)	0.050 (2)	−0.0014 (14)	0.0092 (15)	0.0066 (16)
C3	0.039 (2)	0.034 (2)	0.0418 (19)	0.0042 (15)	0.0063 (15)	0.0018 (15)
C4	0.0251 (16)	0.0293 (18)	0.0404 (16)	−0.0022 (14)	−0.0030 (13)	0.0021 (13)
C5	0.0332 (18)	0.0258 (18)	0.0329 (17)	−0.0015 (13)	−0.0004 (12)	0.0011 (12)
C6	0.0295 (17)	0.0310 (19)	0.0401 (17)	−0.0025 (13)	0.0017 (13)	0.0006 (14)
C7	0.0262 (16)	0.0308 (18)	0.0276 (14)	0.0018 (13)	−0.0005 (13)	−0.0005 (13)
C8	0.0345 (18)	0.0284 (18)	0.0387 (17)	0.0032 (13)	−0.0001 (15)	−0.0023 (14)
C9	0.041 (2)	0.0302 (18)	0.0320 (17)	−0.0054 (14)	−0.0017 (15)	−0.0026 (14)
C10	0.063 (3)	0.027 (2)	0.114 (4)	0.0008 (19)	−0.009 (3)	−0.012 (2)
C11	0.0331 (18)	0.0303 (19)	0.0392 (19)	−0.0076 (14)	0.0003 (13)	0.0005 (13)
C12	0.0366 (19)	0.032 (2)	0.0381 (18)	−0.0019 (15)	0.0003 (14)	−0.0045 (14)
C13	0.0280 (16)	0.0305 (18)	0.0268 (15)	0.0021 (14)	−0.0009 (12)	−0.0016 (12)
C14	0.0259 (16)	0.0297 (18)	0.0256 (15)	0.0010 (13)	−0.0004 (12)	0.0011 (12)
C15	0.0299 (17)	0.040 (2)	0.0350 (16)	−0.0056 (14)	−0.0031 (14)	0.0012 (15)
C16	0.0272 (16)	0.0359 (18)	0.0262 (15)	0.0020 (14)	−0.0018 (12)	−0.0002 (13)
O1S	0.065 (2)	0.0485 (18)	0.0582 (17)	−0.0161 (14)	0.0047 (15)	−0.0012 (14)

### Geometric parameters (Å, º)

**Table:**

O1—H1	0.8400	C5—C6	1.350 (5)
O1—C1	1.362 (4)	C5—C13	1.453 (5)
O2—C6	1.351 (4)	C6—H6A	0.9500
O2—C7	1.371 (4)	C7—C8	1.390 (4)
O3—C9	1.361 (4)	C7—C14	1.396 (4)
O3—C10	1.430 (5)	C8—H8	0.9500
O4—H4	0.8400	C8—C9	1.374 (5)
O4—C12	1.370 (4)	C9—C15	1.401 (5)
O5—C13	1.268 (4)	C10—H10A	0.9800
O6—C16	1.359 (4)	C10—H10B	0.9800
O6—H6	0.87 (4)	C10—H10C	0.9800
C1—C2	1.376 (5)	C11—H11	0.9500
C1—C12	1.402 (5)	C11—C12	1.382 (5)
C2—H2	0.9500	C13—C14	1.430 (4)
C2—C3	1.398 (5)	C14—C16	1.420 (4)
C3—H3	0.9500	C15—H15	0.9500
C3—C4	1.387 (5)	C15—C16	1.366 (5)
C4—C5	1.480 (4)	O1S—H1SA	0.91 (3)
C4—C11	1.402 (5)	O1S—H1SB	0.89 (3)
			
C1—O1—H1	109.5	O3—C9—C8	124.3 (3)
C6—O2—C7	118.6 (2)	O3—C9—C15	113.8 (3)
C9—O3—C10	118.3 (3)	C8—C9—C15	121.9 (3)
C12—O4—H4	109.5	O3—C10—H10A	109.5
C16—O6—H6	104 (3)	O3—C10—H10B	109.5
O1—C1—C2	123.7 (3)	O3—C10—H10C	109.5
O1—C1—C12	116.5 (3)	H10A—C10—H10B	109.5
C2—C1—C12	119.7 (3)	H10A—C10—H10C	109.5
C1—C2—H2	119.9	H10B—C10—H10C	109.5
C1—C2—C3	120.2 (3)	C4—C11—H11	119.6
C3—C2—H2	119.9	C12—C11—C4	120.7 (3)
C2—C3—H3	119.6	C12—C11—H11	119.6
C4—C3—C2	120.8 (3)	O4—C12—C1	116.6 (3)
C4—C3—H3	119.6	O4—C12—C11	123.5 (3)
C3—C4—C5	121.0 (3)	C11—C12—C1	120.0 (3)
C3—C4—C11	118.7 (3)	O5—C13—C5	122.0 (3)
C11—C4—C5	120.3 (3)	O5—C13—C14	121.6 (3)
C6—C5—C4	120.1 (3)	C14—C13—C5	116.4 (3)
C6—C5—C13	118.0 (3)	C7—C14—C13	120.9 (3)
C13—C5—C4	121.9 (3)	C7—C14—C16	116.8 (3)
O2—C6—H6A	117.2	C16—C14—C13	122.3 (3)
C5—C6—O2	125.6 (3)	C9—C15—H15	120.2
C5—C6—H6A	117.2	C16—C15—C9	119.6 (3)
O2—C7—C8	116.3 (3)	C16—C15—H15	120.2
O2—C7—C14	120.4 (3)	O6—C16—C14	119.6 (3)
C8—C7—C14	123.3 (3)	O6—C16—C15	119.4 (3)
C7—C8—H8	121.3	C15—C16—C14	121.0 (3)
C9—C8—C7	117.4 (3)	H1SA—O1S—H1SB	108 (5)
C9—C8—H8	121.3		
			
O1—C1—C2—C3	−179.6 (3)	C5—C13—C14—C16	179.9 (3)
O1—C1—C12—O4	−1.0 (5)	C6—O2—C7—C8	178.0 (3)
O1—C1—C12—C11	179.7 (3)	C6—O2—C7—C14	−2.4 (4)
O2—C7—C8—C9	178.7 (3)	C6—C5—C13—O5	178.9 (3)
O2—C7—C14—C13	2.3 (4)	C6—C5—C13—C14	−0.6 (4)
O2—C7—C14—C16	−178.3 (3)	C7—O2—C6—C5	1.0 (5)
O3—C9—C15—C16	−179.9 (3)	C7—C8—C9—O3	−179.6 (3)
O5—C13—C14—C7	179.8 (3)	C7—C8—C9—C15	0.1 (5)
O5—C13—C14—C16	0.4 (4)	C7—C14—C16—O6	178.7 (3)
C1—C2—C3—C4	0.6 (5)	C7—C14—C16—C15	−0.8 (4)
C2—C1—C12—O4	180.0 (3)	C8—C7—C14—C13	−178.1 (3)
C2—C1—C12—C11	0.7 (5)	C8—C7—C14—C16	1.3 (4)
C2—C3—C4—C5	178.3 (3)	C8—C9—C15—C16	0.3 (5)
C2—C3—C4—C11	−0.5 (5)	C9—C15—C16—O6	−179.5 (3)
C3—C4—C5—C6	−52.6 (5)	C9—C15—C16—C14	0.1 (5)
C3—C4—C5—C13	127.1 (3)	C10—O3—C9—C8	−3.0 (6)
C3—C4—C11—C12	0.5 (5)	C10—O3—C9—C15	177.3 (4)
C4—C5—C6—O2	−179.8 (3)	C11—C4—C5—C6	126.2 (4)
C4—C5—C13—O5	−0.8 (4)	C11—C4—C5—C13	−54.1 (5)
C4—C5—C13—C14	179.7 (3)	C12—C1—C2—C3	−0.7 (5)
C4—C11—C12—O4	−179.8 (3)	C13—C5—C6—O2	0.5 (5)
C4—C11—C12—C1	−0.6 (5)	C13—C14—C16—O6	−1.9 (4)
C5—C4—C11—C12	−178.3 (3)	C13—C14—C16—C15	178.6 (3)
C5—C13—C14—C7	−0.8 (4)	C14—C7—C8—C9	−0.9 (5)

### Hydrogen-bond geometry (Å, º)

**Table:**

*D*—H···*A*	*D*—H	H···*A*	*D*···*A*	*D*—H···*A*
O1—H1···O4^i^	0.84	1.98	2.777 (4)	157
O4—H4···O1*S*	0.84	1.88	2.708 (4)	169
O1*S*—H1*SA*···O5^ii^	0.91 (3)	1.97 (3)	2.855 (4)	164 (5)
O6—H6···O5	0.87 (4)	1.76 (4)	2.577 (4)	155 (4)

Symmetry codes: (i) −*x*, −*y*+1, *z*+1/2; (ii) −*x*+1/2, *y*, *z*−1/2.

## References

[bb1] Bruker (2009). *APEX2* and *SAINT* Bruker AXS Inc., Madison, Wisconsin, USA.

[bb2] Dolomanov, O. V., Bourhis, L. J., Gildea, R. J., Howard, J. A. K. & Puschmann, H. (2009). *J. Appl. Cryst.* **42**, 339–341.

[bb3] Raudnitz, H. & Perlmann, G. (1935). *Ber. Dtsch Chem. Ges. B*, **68**, 1862–1866.

[bb4] Robertson, A., Suckling, C. W. & Whalley, W. B. (1949). *J. Chem. Soc.* pp. 1571–1578.

[bb5] Sheldrick, G. M. (2004). *SADABS* Bruker AXS Inc., Madison, Wisconsin, USA.

[bb6] Sheldrick, G. M. (2008). *Acta Cryst.* A**64**, 112–122. 10.1107/S0108767307043930 18156677

[bb7] Smith, C. T., Noll, B., Waddell, T. G. & Knight, K. S. (2013). *Nat. Prod. Commun.* **8**, 299–300. 23678796

[bb8] Waddell, T. G., Thomasson, M. H., Moore, M. W., White, H. W., Swanson-Bean, D., Green, M. E., Van, H. G. S. & Fales, H. M. (1982). *Phytochemistry*, **21**, 1631–1633.

